# Mood effects on semantic processes: Behavioural and electrophysiological evidence

**DOI:** 10.3389/fpsyg.2022.1014706

**Published:** 2022-11-11

**Authors:** Marcin Naranowicz

**Affiliations:** Faculty of English, Adam Mickiewicz University, Poznań, Poland

**Keywords:** mood, semantic processes, mood induction procedures, processing styles, affective neurolinguistics, lexicosemantic access, N400, LPC

## Abstract

Mood (i.e., our current background affective state) often unobtrusively yet pervasively affects how we think and behave. Typically, theoretical frameworks position it as an embodied source of information (i.e., a biomarker), activating thinking patterns that tune our attention, perception, motivation, and exploration tendencies in a context-dependent manner. Growing behavioural and electrophysiological research has been exploring the mood–language interactions, employing numerous semantics-oriented experimental paradigms (e.g., manipulating semantic associations, congruity, relatedness, etc.) along with mood elicitation techniques (e.g., affectively evocative film clips, music, pictures, etc.). Available behavioural and electrophysiological evidence has suggested that positive and negative moods differently regulate the dynamics of language comprehension, mostly due to the activation of mood-dependent cognitive strategies. Namely, a positive mood has been argued to activate global and heuristics-based processing and a negative mood – local and detail-oriented processing during language comprehension. Future research on mood–language interactions could benefit greatly from (i) a theoretical framework for mood effects on semantic memory, (ii) measuring mood changes multi-dimensionally, (iii) addressing discrepancies in empirical findings, (iv) a replication-oriented approach, and (v) research practices counteracting publication biases.

## Introduction

We experience mood fluctuations of varying intensity, which often subconsciously cloud our judgement and colour our perception. To better understand the complexity of mood effects on cognitive processes, mood changes have been explored in the last three decades mainly from behavioural and electrophysiological perspectives. Overall, most mood research has revolved around two broad categories of a positive and negative mood, consistently pointing to differential cognitive consequences of these two opposite affective states (see Forgas, 2017 for a review). In consequence, various theoretical frameworks have been offered, accounting for and predicting how mood tunes our mental processes. Concurrently, mood researchers have been refining various mood induction procedures (MIPs) – the experimental techniques for ecologically valid and ethically minded mood elicitation under laboratory conditions that reflect mood fluctuations experienced on a daily basis (see Westermann et al., 1996; Lench et al., 2011; Fakhr Hosseini and Jeon, 2017 for reviews).

Growing scholarly attention has been devoted to a potentially reciprocal relationship between mood and language. The mood–language interactions have been explored in various linguistic domains, including syntactic processing (Vissers et al., 2010; Jiménez-Ortega et al., 2012; Van Berkum et al., 2013; Verhees et al., 2015; Liu et al., 2018; Yano et al., 2018), language production (Isen et al., 1985; Beukeboom and Semin, 2006; Kharkhurin and Altarriba, 2016; Hinojosa et al., 2017; Braun et al., 2019; Forgas and Matovic, 2020; Out et al., 2020), communicative interactions (Forgas, 1999; Koch et al., 2013; Matovic and Forgas, 2018), reading patterns (Bohn-Gettler and Rapp, 2011; Scrimin and Mason, 2015; Mills et al., 2019), and emotional word processing (e.g., Kiefer et al., 2007; Pratt and Kelly, 2008; Egidi and Nusbaum, 2012; Kissler and Bromberek-Dyzman, 2021; Naranowicz et al., 2022a). Arguably, semantic processing (i.e., the cognitive mechanisms engaged in language comprehension) has attracted a particularly keen interest among mood researchers, who have employed a variety of behavioural (e.g., Storbeck and Clore, 2008; Sakaki et al., 2011; Matovic et al., 2014) and electrophysiological measures (e.g., Goertz et al., 2017; Ogawa and Nittono, 2019a,b; Naranowicz et al., 2022b) to understand the principles guiding the relationship between our current affective state and how we understand language.

### Defining affective constructs

*Mood* as an affective construct is typically defined through a comparison with *emotion*. Overall, mood and emotion are two affective phenomena differing in duration and intensity, with emotion being characterised as short-lived and rather intense and mood as enduring and mild affective states (Ekman, 1992). Unlike event-triggered full-blown emotion, mood can also be characterised as a diffuse and pervasive background affective state that is rarely associated with a particular object or person (e.g., Elman, 1994; Frijda, 2009). A discrete mood state (e.g., frustration, anxiety, stress, etc.) may still be triggered by an antecedent cause or mood-congruent emotional responses, though (e.g., Morris, 1992; Ekkekakis, 2013). Moreover, the two affective constructs are the products of different appraisal-driven mechanisms: emotions originate from the appraisal of a narrow “adaptational encounter with the environment,” and moods stem from the appraisal of broad “existential issues of one’s life” (Lazarus, 1991: 48). Consequently, whereas the primary function of emotion is to equip us with action packages guiding our immediate behavioural, physiological, and neurological responses in an adaptive fashion (see Nielsen and Kaszniak, 2007 for a review), mood generally tunes our cognitive mechanisms to adapt thinking patterns to our subjective experiences (Schwarz and Clore, 1983). Compared to emotion, mood can also be more strongly affected by interoception (i.e., sensory input from physiological responses or peripheral organs), as reflected by mood fluctuations due to hormones, inflammation, sickness, etc. (Pace-Schott et al., 2019).

Building upon the mood–emotion distinction outlined above, in the present review, I refer to mood as a slowly changing, low-intensity background affective state that mostly unobtrusively fluctuates over time from feeling positive (i.e., pleasant/good) to negative (i.e., unpleasant/bad), with its primary adaptational function being to tune our thinking patterns in a context-dependent manner (see also Forgas, 2017).

### Scope of the present review

The primary aim of this paper is to review accumulating behavioural and electrophysiological research showing how positive and negative moods (i.e., opposite background affective states) modulate the cognitive mechanisms engaged in language comprehension. After outlining the key theoretical frameworks relevant for mood effects on cognitive processing, I focus on ethically-minded methodological aspects of experimental mood elicitation. Next, I review behavioural and electrophysiological research on mood–semantics interactions, considering a range of language comprehension-oriented experimental paradigms. Finally, the paper offers a critical overview of the theoretical and empirical underpinnings of mood–semantic processing interactions, highlighting potential future research directions.

## Theoretical considerations

### Selected theoretical approaches to mood and cognitive processes

Early theoretical frameworks have emphasised highly adaptive motivational consequences of positive and negative moods. First, Clark and Isen (1982) put forward the *affect maintenance* hypothesis, whereby being in a positive mood can subconsciously motivate maintaining such a favourable state of mind by refraining from any effortful, elaborate thinking. In contrast, engagement in vigilant and effortful processing in a negative mood can serve as an adaptive strategy, aiming to improve one’s mood. Then, Schwarz (1990, 2002) developed the *cognitive tuning* hypothesis, suggesting that one’s cognitive processes are regulated by mood to satisfy situational requirements. A negative mood, signalled by negative environmental cues or bodily avoidance feedback (i.e., bodily sensations linked to negative outcomes), is believed to warn us against a problematic situation, motivating a vigilant and effortful processing style. Conversely, a positive mood, signalled by positive environmental cues or bodily approach feedback (i.e., bodily sensations linked to positive outcomes), is assumed to invite reliance on tried and trusted routines, promoting an effortless processing style.

Schwarz and Clore (1983; see also Clore et al., 2001; Clore and Storbeck, 2006) offered a detailed theoretical view on how mood can affect evaluative judgements – the *affect-as-information* (AAI) hypothesis. The AAI hypothesis postulates that affective states (i.e., emotions and moods) are experiential and embodied sources of information about the personal value of whatever is being processed. Emotions and moods reflect unconscious appraisals, typically represented on two orthogonal dimensions of valence (i.e., pleasantness) and arousal (i.e., importance and urgency). The AAI hypothesis assumes misattribution of one’s current mood (i.e., an inferential error) as a response to an object of judgement, leading to its more and less favourable evaluation in a positive and negative mood, respectively. Consequently, positive and negative moods are believed to activate contrastive context-dependent processing styles in problem-solving situations: while a positive mood reinforces top-down relational processing (i.e., relating incoming information to accessible stored information, including knowledge, beliefs, expectations, and stereotypes), a negative mood impedes it, promoting bottom-up referential processing (i.e., focusing on perceptual stimuli from the environment, without associating them with the stored knowledge). In line with the AAI hypothesis, mood is also conceptualised as a marker of task-dependent processing requirements: a positive mood is associated with cognitive ease, motivating effortless and heuristics-based processing, whereas a negative mood signals cognitive difficulty, instigating effortful and systematic processing. The AAI hypothesis also suggests that mood governs available attentional resources: positive and negative moods lead to a global (i.e., top-down) or a local (i.e., bottom-up and detail-oriented) focus of attention, respectively.

Forgas (1995, 2002) proposed an integrative theoretical approach – the *Affect Infusion Model* (AIM) – to account for the control of information processing strategies by mood. The AIM assumes that the intensity of *affective infusion* (i.e., a tendency for thoughts, memories, judgements, and behaviours to be mood-congruent) grows proportionately to context-specific cognitive demands and open information search, giving rise to four distinct processing strategies. First, the *direct access* strategy entails low-effort and automatic retrieval of already stored response that does not require rumination (e.g., retrieving one’s phone number), and it is least impacted by one’s mood. Second, the *motivated processing* strategy involves more effortful yet highly targeted thinking, dictated by a specific motivational objective (e.g., preparing for an exam), and it is minimally affected by one’s mood. Third, the *heuristic processing* strategy concerns effortless subconscious evaluative processing, manifesting itself when such resources as time, interest, attention, motivation, and working memory capacity are in short supply (e.g., choosing an outfit for a party at the last minute). Consistent with AAI hypothesis (Schwarz and Clore, 1983), in such circumstances, one’s affective state can be treated as a heuristic cue, leading to mood-congruent choices. Lastly, the *substantive processing* strategy assumes open and elaborate thinking triggered by a novel and cognitively demanding task in the absence of ready-made solutions (e.g., accidently having to change a career path), leading to the strongest mood-congruent effects.

Building on the AAI hypothesis (Schwarz and Clore, 1983), Bless (2001) proposed the *mood-and-general-knowledge* (MaGK) hypothesis, assuming that mood effects on cognition are associated with one’s reliance on general knowledge structures. The MaGK hypothesis holds that experiencing a positive mood signals being in a benign situation, which consequently promotes reliance on pre-existing general knowledge structures and a heuristics-based, top-down thinking style. Conversely, experiencing a negative mood may be associated with eminent threat, motivating a more analytical, detail-oriented, bottom-up thinking style. The MaGK hypothesis also assumes that reliance on general knowledge structure in a positive mood saves up cognitive resources that can be allotted to other cognitive tasks and leads to making inferences beyond the information given (e.g., to form false memories).

Bless and Fiedler (2006) offered the *adaptive function* account – another theoretical perspective on how mood influences information processing styles. This account stipulates that, when attention drives thinking, mood effects on thinking styles are dictated by two complementary biological tendencies: *assimilation* (i.e., modifying new information to fit into internal structures) and *accommodation* (i.e., modifying internal structures to fit into new information). In line with the adaptive function perspective, a positive mood promotes an assimilative (i.e., schema-based and top-down) thinking style and a negative mood an accommodative (i.e., externally focused and bottom-up) thinking style.

Zadra and Clore (2011) put forward an alternative *bio-energetic* perspective on how mood alters cognitive processes. They proposed that mood serves as a biological marker of the number of resources that can be readily invested in exploratory (i.e., cognitively intense) behaviour. Specifically, affective states are assumed to involuntarily provide embodied information about energy costs and likely benefits of potential actions, acting in the interest of resource maintenance. Exploratory and exploitatory behaviours are therefore promoted by a positive and negative mood, respectively. Consistently with the AAI hypothesis (Schwarz and Clore, 1983), the bio-energetic perspective also capitalises on the attention-mediated global–local attentional focus, with a positive mood broadening the scope of attention (i.e., a global perceptual style) and a negative mood narrowing it (i.e., a local perceptual style). Moreover, the bio-energetic perspective highlights the role of arousal, whose main function is to direct available processing resources to the most significant (i.e., the most arousing) information.

Herz et al. (2020; see also Bar, 2021) developed the *State of Mind* (SoM) framework, offering a more holistic approach to one’s psychological state of mind, emphasising the role of mood in its regulation. The SoM framework capitalises on an overarching and dynamic construct termed *state of mind* (i.e., one’s current behavioural, cognitive, and affective inclinations) regulated by five inter-related dimensions of perception (sensory information vs. predictions), attention (global vs. local), thought (broad vs. narrow thinking style), openness to experience (exploration vs. exploitation), and affect (a positive vs. negative mood). All of the dimensions are assumed to change together in a synchronised manner, and their role is dictated by a varying ratio of bottom-up (i.e., a broad SoM) to top-down (i.e., a narrow SoM) cortical processing. Consistent with the SoM framework, being in a positive mood entails more bottom-up processing, accompanied by greater reliance on sensory information, a global focus of attention, a broad thinking style, and exploratory disposition. In contrast, being in a negative mood involves more top-down processing and, consequently, greater reliance on predictions, a local focus of attention, a narrow thinking style, and exploitatory disposition. Strikingly, while the “bottom-up” and “top-down” types of processing have consistently been associated with negative and positive mood, respectively, by other theoretical frameworks (e.g., Schwarz and Clore, 1983), Herz et al. (2020) propose the opposite mapping between processing styles and positive and negative moods.

### Selected theoretical approaches to mood and language comprehension

Mood has also been incorporated into a recent theoretical model of language comprehension. Operating at the intersection of pragmatics, psycholinguistics, and affective research, van Berkum et al. (2018; see also Van Berkum, 2019) proposed the *Affective Language Comprehension* (ALC) model, emphasising the role of affect in language comprehension during communicative interactions. The first stage of the comprehension process entails activating and retrieving lexical, semantic, phonological, and syntactic representations of individual items from long-term memory, which are later pieced together and comprehended as a whole. The verbal message is communicated alongside non-verbal cues (e.g., gestures, gaze, facial expressions, etc.). The second stage of the process involves inferring (i.e., interpreting) the conventionalised meaning communicated by the speaker: their referential intention (i.e., to whom a given message refers), their stance (i.e., certainty as well as conscious or unconscious affective/evaluative orientation), their social intention (i.e., whether they intend to share, request, or inform about something), and bonus meaning (i.e., what can be inferred beyond the communicated meaning). The ALC model stresses the importance of *emotionally competent stimuli* (i.e., stimuli automatically triggering emotions), which can affect *all* individual processes involved in language comprehension, also accounting for such affective qualities as empathy, emotional contagion, empathetic concern, and affective perspective-taking. Crucially, the ALC model acknowledges that mood tunes cognitive processes, recognising the mediating role of the recipient’s mood in interpreting the conveyed message, and that emotionally evocative language itself may elicit a given mood. Yet, no mood-dependent cognitive consequences for language comprehension are stipulated by the model.

## Methodological considerations

### Mood induction procedures

Various MIPs have been employed by psycholinguists to experimentally manipulate participants’ mood. Typically, participants are exposed to well-controlled affectively evocative stimuli (e.g., film clips, music, pictures, written stories, self-referential statements, etc.) under laboratory conditions so as to temporarily alter and/or intensify their current affective states. Most experimental studies involving mood elicitation concentrate on two broad categories of positive and negative moods, assumed to reflect the mood fluctuations experienced in everyday life (see Westermann et al., 1996; Lench et al., 2011; Fakhr Hosseini and Jeon, 2017; Joseph et al., 2020 for reviews). In practice, a medium- or high-intensity pleasant or unpleasant affective state is elicited through repeated and rather intense exposure to stimuli charged with either positive or negative emotions, effects of which are expected to cumulate and affect the cognitive processes of interest throughout the entire experiment. Some mood researchers also incorporate a more elusive category of a “neutral” mood into their research designs: a baseline condition representing a low-intensity calming affective state, elicited through presentation of equivalent yet affectively neutral stimuli (e.g., a nature documentary instead of an affectively rich film fragment) or no manipulation (see Fernández-Aguilar et al., 2019 for a review).

Substantial evidence has pointed to greater emotional reactivity to negative compared to positive mood induction. In their systematic review, Joseph et al. (2020) estimated that on average negative content exerts nearly two times stronger mood changes than positive content, as indexed by self-reports. This finding is consistent with the negativity bias hypothesis (see Norris, 2021 for a review), whereby negative relative to positive events generally have greater impact on our cognitive state (e.g., behaviour, perception, decision making, physiology, attention, etc.) due to different adaptive functions of positive and negative affective states. Namely, negative affective states signal the presence of a stimulus threatening one’s homeostatic balance, thereby eliciting survival-driven physiological and cognitive responses, which is not the case for positive affective states (Baumeister et al., 2001). It is also noteworthy that since participants typically are in a mildly positive mood upon arrival to the laboratory (Joseph et al., 2020), lack of a significant increase in mood ratings in the positive mood condition is not perceived as ineffective mood manipulation when participants maintain the targeted positive mood (e.g., Van Berkum et al., 2013).

Elicitation of positive and negative moods *via* film clips deserves special attention due to its highest potency among MIPs (Westermann et al., 1996; Joseph et al., 2020) and its prevalence in psycholinguistic research (e.g., Hänze and Hesse, 1993; Bless et al., 1996; Chwilla et al., 2011; Van Berkum et al., 2013; Vissers et al., 2013; Matovic et al., 2014; Goertz et al., 2017; Naranowicz et al., 2022a,b). Such affectively charged audio-visual materials have been favoured due to their ability to create affectively dynamic contexts that reflect real-life situations (i.e., they have high ecological validity; Fernández Megías et al., 2011), motivating high attentional engagement (Rottenberg et al., 2007), and eliciting the affective states lasting for exploitable lengths of time (Carvalho et al., 2012). From a practical perspective, a number of databases offering standardised and validated mood-inducing film clips have been developed (see Maffei and Angrilli, 2019), targeting both general (i.e., positive and negative moods) and discrete affective states (e.g., sadness, joy, fear, disgust, etc.). Though there is no clear consensus over the most desirable characteristics of mood-inducing film clips (e.g., genre, duration, brightness, etc.), Maffei and Angrilli (2019) estimated that 2 min is the optimal duration for film clips in terms of the effectiveness in inducing targeted moods and participants’ engagement.

To intensify the mood effects elicited with film clips, some researchers have recommended (i) explicitly informing participants about the purpose of mood induction (Westermann et al., 1996), (ii) instructing participants to put themselves in the targeted mood (Rottenberg et al., 2018), (iii) asking participants to imagine themselves as one of the protagonists, and (iv) sympathising with other characters (Werner-Seidler and Moulds, 2012). A similar conclusion could also be drawn from a recent meta-analysis by Joseph et al. (2020), who revealed that affectively evocative films indeed exert the strongest effects on participants’ affective states among all MIPs when the intent of mood induction is truthfully revealed to them. It remains an open question, however, if being fully aware of the fact that the affective context is created experimentally increases participants’ emotional reactivity or promotes following demand characteristics (see the *Measuring mood changes* section below for more details).

### Ethical considerations

In real life, deliberate manipulation of one’s affective state has many facets and is generally considered immoral, especially when it involves deceptive and underhanded tactics. While triggering increased affective reactions remains a part and parcel of experimental mood research and is not readily perceived as manipulative, it remains critical to predict and minimise its potential negative consequences (Fakhr Hosseini and Jeon, 2017). Occurrences of mood disorders (e.g., clinical depression, bipolar disorder, borderline personality disorder, etc.) and recent traumatic events (e.g., a close relative’s death) in prospective participants are the two ethical challenges particularly problematic in the case of negative mood induction. Exposure to such destressing content can seriously destabilise emotional well-being of vulnerable individuals. For instance, individuals with depression exposed to negative content are likely to excessively ruminate on their own symptoms, which typically exacerbates a negative mood and predicts depressive episodes (Joormann and Stanton, 2016). Potential participants should therefore be explicitly informed about the deeply emotional character of the mood-inducing stimuli beforehand, with particular attention devoted to increased risks to vulnerable individuals. Pre-screening procedures could also include a standardised psychometric test for common mood disorders (e.g., DASS-21 measuring one’s level of depression; Lovibond and Lovibond, 1995), which can help identify individuals with undiagnosed mood disorders as well as those reluctant to openly report suffering from them in a medical history questionnaire. Moreover, to facilitate emotional recovery post negative mood induction, participants should be exposed to mood “reset” induction (e.g., evoking good memories from the past while listening to positive music; Joseph et al., 2020).

### Measuring mood changes

Traditionally, experimentally evoked mood changes have been measured with self-report inventories, administered prior to, in-between, and post mood induction phases (see Gray and Watson, 2007; Ekkekakis, 2013 for reviews). Mood self-assessment is a metacognitive and introspective undertaking: participants are expected to identify and interpret elusive physiological sensations, take into account the surrounding context, and quantify all of these factors using rating scales (Gray and Watson, 2007).

The choice of a mood measure most informative in a given study could depend on whether one conceptualises mood as positioned along orthogonal dimensions or as a blend of distinct affective states, which aligns with conventional theoretical approaches to affective constructs (see Ekkekakis, 2013 for a review). First, participants are frequently asked to measure their current mood on two bipolar scales of mood valence (i.e., positive––negative) and arousal (i.e., low––high; e.g., Sakaki et al., 2011; Vissers et al., 2013; Wang et al., 2016). This is consistent with the *dimensional approach* to affect (e.g., Russell, 1980), positing that affective states can be represented along two orthogonal (i.e., unrelated) and bipolar dimensions of valence/pleasantness (i.e., an evaluative/hedonic component) and arousal/activation (i.e., an intensity component), the combination of which captures affective states. An alternative to two bipolar scales is the use of two separate unipolar scales of a positive mood (or happiness) and a negative mood (or sadness; e.g., Hesse and Spies, 1996; Bolte et al., 2003). While it is theoretically possible, such an approach does not assume that one concurrently experiences the two affective states at intense levels. In fact, positive and negative mood (or happiness and sadness) ratings post mood induction are usually negatively correlated: an increase in a positive mood/happiness is accompanied by a decrease in a negative mood/sadness, and vice versa (e.g., Scollon et al., 2005; Brehm and Miron, 2006; Joseph et al., 2020). Overall, the dimensional approach could be particularly informative in research taking a more holistic perspective on mood, that is, focusing on general positive and negative mood states.

Second, participants can also be asked to assess their current affective state by rating it on numerous unipolar scales, represented by mood-related state adjectives (e.g., Pinheiro et al., 2013; Van Berkum et al., 2013). Pinheiro et al. (2013), for instance, administered the Profile of Mood States questionnaire (PoMS; McNair et al., 1971): participants rated their current affective state using 65 state adjectives (e.g., *friendly, tense, angry,* etc.) or simple statements (e.g., *sorry for things done*, *ready to fight*, etc.). The ratings were then grouped into seven subcategories indexing different mood states (i.e., *depression, tension, anger, vigour, fatigue, confusion,* and *friendliness*). This is consistent with the *distinct-state approach* (also known as a *categorical approach* or *state-affect approach*) to affective constructs (e.g., Izard, 1993), positing that our current mood is a composite of unique and unrelated affective states that are believed to solve unique adaptive problems. The distinct-state approach seems to be most suitable for research on individual mood states (e.g., anxiety or tension); however, it has still been adopted in research on general positive and negative moods (e.g., Pinheiro et al., 2013; Van Berkum et al., 2013) to tap into a more detailed picture of experimentally induced mood states.

Despite being widely embraced by mood researchers, self-report mood questionnaires can be subject to a number of random and systematic measurement errors (see Gray and Watson, 2007 for a review). One of the potential threats to reliable mood assessment under laboratory conditions relates to the social desirability bias: a respondent’s tendency to inaccurately answer socially sensitive questions, such as those related to their affective state, so as to be perceived in a more favourable light by others (Ekkekakis, 2013). Self-report mood measurements are also subject to demand characteristics: a respondent’s tendency to behave in a manner they believe is expected of them (Fakhr Hosseini and Jeon, 2017). For instance, having watched a number of sad film clips or been informed about the purpose of mood induction (see the *Mood induction procedures* section above), participants may assume that they are expected to report a deterioration in their mood state, purposefully downgrading their mood ratings. Another potential problem lies in some people’s inherent inability to identify or interpret physiological indicators of their affective states on top of countless other external factors influencing it, thereby leading to much variability in mood ratings. Nielsen and Kaszniak (2007) proposed that those participants who are more emotionally aware are better emotion regulators, and those who underwent an emotion-related formal training may be more aware of their affective experiences than others. Gray and Watson (2007), in turn, observed that, unlike “high awareness” participants, those insensitive to changes in their affective state may rely on cultural and gender stereotypes when rating their current mood (e.g., see the *Blue Monday* and *Thank God it’s Friday* effects; Stone et al., 2012).

## Mood effects on semantic processes

### Behavioural evidence

Growing behavioural evidence has indicated that positive and negative moods may exert marked effects on semantic processes (e.g., Hänze and Hesse, 1993; Bless et al., 1996; Hesse and Spies, 1996; Bolte et al., 2003; Rowe et al., 2006; Storbeck and Clore, 2008; Sakaki et al., 2011; Matovic et al., 2014; see also Supplementary materials). Much behavioural research has concentrated on how mood affects spreading activation in semantic memory employing a semantic priming paradigm, wherein participants are presented with semantically (un)related prime–target word pairs (Hänze and Hesse, 1993; Hesse and Spies, 1996; Storbeck and Clore, 2008). In such a paradigm, researchers typically observe a so-called *semantic priming effect*: reduced response latencies for a target word (e.g., *dog*) preceded by a semantically related (e.g., *cat*) compared to an unrelated prime word (e.g., *car*), which is believed to reflect facilitated spreading activation of semantically related concepts (Hänze and Hesse, 1993). Semantic priming has been employed in combination with a lexical decision task (LDT; i.e., classifying a string of letters as a word or a non-word; Hänze and Hesse, 1993; Hesse and Spies, 1996; Storbeck and Clore, 2008) and a semantic categorisation task (SCT; i.e., judging semantic relatedness of presented category–member pairs; Storbeck and Clore, 2008; *cf.* Sakaki et al., 2011). Moreover, the organisation of semantic memory has also been explored through a remote association paradigm, wherein participants are typically presented with three words (i.e., word triads) somewhat semantically (dis)associated with a common fourth word and make intuitive judgements about their semantic coherence (Bolte et al., 2003; Rowe et al., 2006). Moreover, a remote association paradigm combined with perceptual tasks have also been used to test how mood influences attentional focus. Finally, some attention has also been devoted to the question of how mood affects reliance on general knowledge structures (i.e., heuristics), explored through the manipulation of information typicality and relevance (Bless et al., 1996).

As for a positive mood, behavioural research employing a semantic priming paradigm has suggested that it may facilitate spreading activation to close but not remote associates (i.e., the words of high/low semantic associations, respectively) in semantic memory (Hänze and Hesse, 1993). In an LDT, Hänze and Hesse (1993) explored how film-induced positive and neutral moods modulate semantic priming, manipulating the associative strength (high vs. low) of the prime–target pairs. They observed stronger semantic priming for the prime–target pairs of high but not low associative strength in a positive mood only, pointing to positive mood-driven improved spreading activation to closely associated concepts. Hänze and Hesse (1993) also concluded that such a facilitatory effect of a positive mood on the activation level may decrease proportionally to decreasing activation strength between two neighbouring concepts.

Still, facilitated activation spread to remote associates in semantic memory in a positive mood has actually been found in behavioural research employing a remote association paradigm (Bolte et al., 2003). Bolte et al. (2003) investigated how positive, negative, and neutral moods elicited by autobiographical recall alter intuitive semantic coherence judgements (i.e., their accuracy, duration, and confidence in the decisions made) about word triads weakly associated with a fourth word. They observed that the intuitive coherence judgements were more accurate in a positive relative to neutral and negative mood, with a negative mood leading to coherence judgements only at chance level. Bolte et al. (2003) proposed that a positive mood may promote and a negative mood may restrict the activation of widespread associative networks in semantic memory, linking such patterns with adapting mood-dependent cognitive strategies.

Further behavioural research employing a remote association paradigm has also indicated that a positive mood may result in increased breadth of attentional selection, facilitating cognitive processes that require a global attentional focus (e.g., semantic processing), at the same time impairing those that require a narrow attentional focus (e.g., perceptual processing; Rowe et al., 2006). In a series of semantic and perceptual experiments, Rowe et al. (2006) tested how music-induced positive, negative, and neutral moods affect intuitive coherence judgements in a remote association paradigm as well as visual selective attention, using strings of compatible letters or with one incompatible letter. They found that a positive compared to negative and neutral mood provoked increased generation of semantically distant associations, indicating a broader attentional focus triggered by a positive mood. They also observed slower RTs to incompatibility trials in a positive compared to negative and neutral moods, pointing to a potential adverse effect of a positive mood on selective attention.

Moreover, behavioural research has also suggested that a positive mood, associated with increased cognitive ease, may promote reliance on general knowledge structures (i.e., heuristics) to a greater extent than neutral and negative moods (Bless et al., 1996). In three semantic and perceptual experiments, Bless et al. (1996) investigated how positive, negative, and neutral moods (elicited through autobiographical recall and films) alter the recognition speed and accuracy of critical words (un)related to auditorily-presented stories, varying in information typicality and relevance. They found a stronger tendency among participants to erroneously classify typical in contrast to atypical and irrelevant information as related to a given story (i.e., an intrusion error) in a positive compared to negative mood, with a neutral mood falling in-between. Bless et al. (1996) suggested that such reliance on pre-existing knowledge in a positive mood could not result from decreased processing capacities or motivation, as a positive compared to neutral and negative mood also facilitated response accuracies in a secondary concentration task (i.e., identification of certain physical attributes of letters).

Regarding a negative mood, previous behavioural research employing semantic priming has pointed to its inhibitory effects, translated into dampened activation of semantic associations in semantic memory (Storbeck and Clore, 2008). Note that such a finding has also been corroborated by research employing a remote association paradigm (Bolte et al., 2003). Storbeck and Clore (2008) studied how music-induced positive, negative, and neutral moods influence semantic priming in an LDT and an SCT as well as affective priming (i.e., faster RTs to a target word affectively congruent relative to incongruent with a prime) in an evaluative task (i.e., classifying words as positive or negative). To this aim, they used the prime–target pairs varying in word status (i.e., words vs. non-word), semantic categories (i.e., animal- vs. texture-related), and word valence (i.e., positive vs. negative), respectively. They observed semantic and affective priming effects in positive and neutral moods, with no such effects in a negative mood, suggesting that a negative mood may actually result in impaired spreading activation in semantic memory. Also, Storbeck and Clore (2008) suggested that such results do not contradict the previously observed facilitatory effect of a positive relative to neutral mood on semantic priming, given that their participants in the neutral (i.e., baseline) mood condition had in fact reported being in a mildly positive mood.

Further behavioural research has offered corroborative evidence for deleterious effects of a negative mood on spreading activation in semantic memory (Bolte et al., 2003; Storbeck and Clore, 2008), additionally indicating that a negative mood may at the same time leave perceptual processing intact (Sakaki et al., 2011). In three semantic categorisation and perceptual experiments, Sakaki et al. (2011) examined how picture-induced positive, negative, and neutral moods alter the speed and accuracy of binary semantic judgements about (un)related word pairs in an SCT as well as binary perceptual judgements (i.e., judging the colour/shade of individual letters). Overall, Sakaki et al. (2011) observed slower RTs in SCTs in the negative compared to positive and neutral mood conditions, pointing again to an inhibitory effect of a negative mood on activation spread in semantic memory. They also suggested that the observed task-dependent differences may point to a negative mood interfering with the activation of verbal working memory, imposing higher cognitive demands. Yet, there were no between-mood differences in the perceptual tasks, irrespective of their difficulty, indicating no behavioural mood effect on attention.

In contrast, behavioural research employing semantic priming has also suggested that a negative mood may actually promote systematic (i.e., structured) semantic associations among the concepts in semantic memory (Hesse and Spies, 1996). In an LDT, Hesse and Spies (1996) tested how Velten sentences–induced (i.e., reading and contemplating over self-referential affirmatives evoking a targeted mood state) and music-induced negative and neutral moods influence semantic priming. Besides non-words, they used structured (i.e., based on synonyms and type–token relations), unstructured (i.e., based on idiomatic speech relations), and unrelated prime–target pairs. They also employed a longer stimulus onset asynchrony (SOA) of 500 ms, as it is believed to facilitate controlled and not automatic word processing. Hesse and Spies (1996) found a larger semantic priming effect for the well-structured prime–target pairs in a negative compared to neutral mood, with no between-mood effect for the unstructured pairs. They concluded that a negative mood may direct attention to structured (i.e., systematic) semantic relations between words, facilitating their activation in semantic memory.

Behavioural research has also indicated that a negative mood may promote greater attention to detail and an accommodative processing style (Matovic et al., 2014). Using a free recall paradigm, Matovic et al. (2014) tested how film-induced positive, negative, and neutral moods alternate the speed and accuracy of binary and rating judgements of the clarity of (un)ambiguous sentence pairs and their free recall. They found that the ambiguities were discriminated slower yet with greater precision in a negative compared to positive and neutral mood, with more information also being recalled in a negative compared to positive mood. Of note, a positive mood did not mirror the results observed in a negative mood, and there was no difference between positive and neutral moods.

Finally, behavioural research has also shown that a negative mood may impede predictive sentence processing to a greater extent in older than younger adults (Liu, 2021). Employing a self-paced reading paradigm, Liu (2021) explored how music-induced positive and negative moods affect the accuracy of binary comprehension judgements along with RTs to critical words embedded in highly and lowly predictable sentences in younger (*M_Age_* = 19.7 years) and older adults (*M_Age_* = 65.9 years). They found that while both younger and older adults effectively discriminated between highly and lowly predictable sentences in a positive mood, such an effect was observed only for younger adults in a negative mood, suggesting that a negative mood impedes language comprehension in older individuals.

### Electrophysiological evidence

Accumulating electrophysiological evidence has also pointed to marked mood effects on semantic processes (Chung et al., 1996; Federmeier et al., 2001; Chwilla et al., 2011; Jiménez-Ortega et al., 2012; Pinheiro et al., 2013; Van Berkum et al., 2013; Vissers et al., 2013; Wang et al., 2016; Goertz et al., 2017; Ogawa and Nittono, 2019a,b; Naranowicz et al., 2022b; see also Supplementary materials), yet employing different experimental paradigms than behavioural research. Two ERP components indexing semantic processing have been observed to be particularly sensitive to mood fluctuations: the N400 and the P600 or late positive complex (LPC). The N400 is a negative-going brainwave with a centroparietal scalp distribution and slight right-hemisphere bias, peaking in amplitude between 300–500 ms post stimulus onset (Kutas and Hillyard, 1980). The N400 is typically responsive to semantic violations: more pronounced N400 amplitudes are observed in response to critical words semantically incongruent with a given context (e.g., Moreno and Kutas, 2005), semantically congruent yet implausible in a given context (e.g., Kutas and Hillyard, 1984), and incongruent with one’s general world knowledge (e.g., Kuperberg et al., 2003). A linear decline in N400 amplitudes indexes the activation of more predictive mechanisms (i.e., greater expectedness) and, thereby, fewer cognitive resources engaged in lexicosemantic access. This leads to less effortful and, consequently, faster retrieval of word meanings from long-term memory (Kutas and Hillyard, 1980). The LPC (also known as the “semantic P600”) is a positive-going brainwave, typically with a parietal scalp distribution and slight left-hemisphere bias, peaking in amplitude at around 500–900 ms (Friedman and Johnson, 2000). Besides its sensitivity to syntactic violations (i.e., “syntactic P600”; Hagoort et al., 1993), the P600/LPC is also responsive to semantic incongruities and expectancies (e.g., Spotorno et al., 2013), with higher P600/LPC amplitudes mirroring the mechanisms engaged in re-analysis and integrating the information retrieved from long-term memory with a broader context (Brouwer et al., 2012).

Early electrophysiological research has suggested that a mild positive mood may facilitate lexicosemantic access to distantly related concepts in semantic memory, at least in females (Federmeier et al., 2001). In a passive reading task, Federmeier et al. (2001) tested how picture-induced mild positive and neutral moods influence the comprehension of sentence pairs with embedded expected words (EWs), within-category violations (WCVs; i.e., unexpected words of the same semantic category), and between-category violations (BCVs; i.e., unexpected words of a different yet semantically-related category). They observed that BCVs elicited the most pronounced N400 amplitudes, followed by WCVs, and finally EWs in a neutral mood. In a positive mood, BCVs elicited a reduced N400 response, eliminating the differences between the two types of violations. Such a pattern thus points to facilitation of lexicosemantic access to distantly related concepts. Crucially, given that the faciliatory effect of a positive mood occurred only in female and not male participants, gender might be a potential moderator of the mood–language interactions (see also Naranowicz et al., 2022a).

In contrast, further electrophysiological evidence has pointed to a positive mood accelerating lexicosemantic access to closely related concepts and a negative mood inhibiting lexicosemantic access to weakly related concepts in males (Pinheiro et al., 2013). Similarly to Federmeier et al. (2001), Pinheiro et al. (2013) studied the relationship between picture-induced positive, negative, and neutral moods and semantic processing in a semantic decision task (SDT; i.e., classifying sentences as meaningful or meaningless), employing EWs, WCBs, and BCWs and focusing on males only. In a neutral mood, Pinheiro et al. (2013) observed a graded effect, with the highest N400 amplitudes evoked by BCVs, followed by WCVs, and finally EWs, similarly to Federmeier et al. (2001). In a positive mood, the N400 amplitudes elicited by EWs and WCVs converged and were both lower than for BCVs, suggesting a positive mood-driven facilitation of lexicosemantic access the words from the same semantic category. In a negative mood, the N400 amplitudes elicited by BCVs and WCVs converged and were both higher than for EWs, pointing to a negative mood-driven impairment of lexicosemantic access to the words belonging to different semantic categories. Pinheiro et al. (2013) also observed attenuated N400 responses to EW in a negative compared to positive mood, suggesting that a negative mood may promote the generation of narrowed predictions that may sensitise us to the most relevant contextual information yet not to the relationship between different concepts in semantic memory.

Other electrophysiological evidence has also pointed to qualitative differences in positive and negative mood effects on semantic processing (i.e., mood-dependent processing), instead of the activation of mood-driven facilitatory or inhibitory mechanisms (Chwilla et al., 2011). In a passive reading study, Chwilla et al. (2011; see also Dwivedi and Selvanayagam, 2021 for corroborative evidence regarding dispositional affect) investigated the effects of film-induced positive and negative moods on the comprehension of neutral sentences, containing high- and low-cloze words (i.e., highly expected and rather unexpected words, respectively). They found an attenuated N400 cloze probability effect (i.e., a difference in N400 amplitudes between high and cloze probability conditions) in the negative compared to positive mood condition: while the N400 effect was broadly and bilaterally distributed in a positive mood, it was constrained to the right hemisphere and the left occipital and temporal sites in a negative mood. The N400 effect size correlated positively with participants mood ratings. The results indicate that, instead of facilitating/hindering meaning-related cognitive processes (e.g., motivation or attention), mood may lead to qualitatively different processing strategies, activating heuristics-based and detail-oriented processing modes in a positive and negative mood, respectively. Additionally, low- relative to high-cloze probability sentences elicited more pronounced P600/LPC amplitudes in a negative mood only, suggesting that semantically anomalous information is re-analysed probably due to a negative mood triggering local, detail-oriented processing.

Some electrophysiological research has also shown that mood effects on lexicosemantic access may be dependent on the allocation of attentional resources, with a positive mood triggering selective attention to the most relevant information and a negative mood non-selective attention to all semantic relations (Wang et al., 2016). Combining a passive reading task with an SDT, Wang et al. (2016) looked into how odour-induced positive and negative mood regulated the processing of question-answer pairs, manipulating their semantic congruity (i.e., whether critical words were semantically congruent with the question context) and task-relevance (i.e., whether critical words were relevant to questions or not). They found that while incongruent words elicited larger N400 amplitudes than congruent ones regardless of task-relevance in a negative mood, such an N400 congruity effect was observed only for task-relevant words in a positive mood. These results can be accounted for by a mood-triggered attentional shift during lexicosemantic access: while language users experiencing a positive mood seem to allocate their attentional resources to the most relevant contextual information, a negative mood may trigger non-selective and analytical information processing, directing equal attention to semantic relations among all words, regardless of whether they are critical to a given context or not.

Electrophysiological evidence has also suggested that a positive compared to negative mood may promote reliance on general knowledge structures (i.e., heuristics), leading to increased cognitive effort invested in semantic integration and re-evaluation (Vissers et al., 2013). Vissers et al. (2013) tested how film-induced positive and negative moods influence the processing of semantically plausible and implausible (i.e., conflicting with general world knowledge) sentences. Though they observed no N400 modulations by mood, implausible sentenced elicited larger P600/LPC amplitudes than plausible sentences in a positive but not in a negative mood. The P600/LPC effect size correlated positively with participants mood ratings. With no mood-dependent differences during the lexicosemantic access stage (indexed by N400 responses), these results again point to the activation of mood-dependent processing modes during semantic re-analysis, with a positive mood reinforcing global heuristics-based processing and a negative mood promoting local detail-oriented one. An alternative explanation offered by Vissers et al. (2013) was that people in a positive mood may be more attentive to semantic anomalies and/or better motivated than those in a negative mood. Interestingly, they also found a left-lateralised effect contrasting with the P600 (i.e., an anterior negativity) in a negative mood only, suggesting that a negative mood may increase working memory demands.

Recent electrophysiological research has offered corroborative evidence for reliance on heuristics in a positive mood during meaning integration, additionally pointing to similar mood-driven mechanisms being activated during native (L1) and non-native (L2) language processing (Jankowiak et al., 2022). In an SDT, Jankowiak et al. (2022) explored how film-induced positive and negative moods influence creative meaning processing in proficient Polish–English bilinguals, presenting participants with words embedded in literal, anomalous, and novel metaphoric sentences. Unlike in Naranowicz et al. (2022b), the anomalous sentences were built based on general knowledge violations. Jankowiak et al. (2022) observed expected higher P600/LPC amplitudes to anomalous compared to both novel metaphoric and literal sentences in a positive mood, suggesting that general knowledge violations required increased semantic integration and re-analysis, irrespective of language of operation. Yet, there were no differences in P600/LPC responses between the three sentence types in a negative mood, suggesting that a negative mood may promote more attentive and detail-oriented processing, decreasing reliance on heuristics.

Similarly, other electrophysiological evidence has also suggested that a negative mood may impede heuristics-based anticipatory mechanisms (Van Berkum et al., 2013). In a passive reading experiment, Van Berkum et al. (2013) researched film-induced positive and negative mood effects on referential anticipation employing short stories with bias-consistent (i.e., confirming) and bias-inconsistent (i.e., disconfirming) expectations about pronouns referring to a first- or second-mentioned character. They found that bias-consistent relative to bias-inconsistent pronouns elicited a larger ERP positivity in the 400–600 ms time window in a positive mood, with no such an ERP pattern in a negative mood. These results evince that a negative mood may impede associative retrieval from long-term memory, possibly mediated by increased inhibitory control. Alternatively, the results may also be accounted for through a bio-energetic perspective (Zadra and Clore, 2011), whereby a negative mood may hinder exploratory behaviour, including some aspects of meaning-related anticipatory processes (e.g., referential anticipation).

Finally, electrophysiological research on the mood–language interactions has recently been extended to the bilingual context (Kissler and Bromberek-Dyzman, 2021; Naranowicz et al., 2022b; Jankowiak et al., 2022; see also Naranowicz et al., 2022a), demonstrating that positive and negative moods may differently affect consecutive stages of L1 and L2 processing (Naranowicz et al., 2022b). In an SDT, Naranowicz et al. (2022b) explored how film-induced positive and negative moods affect bilingual language processing in Polish–English bilinguals, who made meaningfulness judgements on words embedded in meaningful (i.e., expected) and meaningless (i.e., rather unexpected) sentences. First, Naranowicz et al. (2022b) observed that a positive mood may lead to increased attentional focus, irrespective of language of operation, as indexed by higher P1 (i.e., a marker of pre-lexical perceptual processing modulated by attention) amplitudes in a positive compared to negative mood. Second, they also found that a negative mood may promote detail-oriented processing of lexical information in a language requiring in a given moment higher cognitive demands. This was marked by two mirrored ERPs patterns: a reduced N1 (i.e., a marker of early lexical access) response in a negative compared to positive mood in L2 only together with a reduced N2 (i.e., a marker of early lexicosemantic processing) response in a negative compared to positive mood in L1 only. Third, Naranowicz et al. (2022b) also found a facilitatory effect of a positive mood on lexicosemantic processing, yet only in the L1 context. This was indexed by an increased N400 response to meaningless compared to meaningful sentences in a positive mood in L2, with no such a difference in L1 in a positive mood. Finally, they also found that a negative mood may temporarily suppress full semantic integration of L2 content, likely to “protect” bilinguals from adverse effects of a negative mood (see Wu and Thierry, 2012). This was marked by an increased P600/LPC response to L2 than L1 meaningful sentences in a negative mood only.

## General discussion

### Theoretical considerations

Theoretical modelling has a high epistemic value, providing researchers with explanatory insights into observable phenomena. The above reviewed theoretical accounts delineating mood effects on cognitive mechanisms have conjured up a complex yet rather consistent picture. Overall, such frameworks predict that mood functions as a biological marker – an embodied source of information about one’s current state of mind, activating context-dependent cognitive strategies (i.e., mood-dependent processing). Therefore, its adaptational role is to help us adapt our behaviour in socially complex situations by tuning numerous cognitive mechanisms.

The reviewed theoretical models have together revealed that mood may affect four different cognitive faculties: perception, attention, motivation, and exploration tendencies. Crucially, research on the mood–language interactions has offered some empirical support for some of them. First, mood has been hypothesised to modulate *perception*: positive and negative moods may, respectively, increase reliance on already stored general knowledge (i.e., heuristics-driven, assimilative and relational thinking) and analysis of environmental stimuli (i.e., accommodative and referential thinking; e.g., Schwarz and Clore, 1983; Bless, 2001; Bless and Fiedler, 2006; *cf.* Herz et al., 2020). Such predictions have gained support in both behavioural (Bless et al., 1996) and electrophysiological research on the mood–semantics interactions (Van Berkum et al., 2013; Vissers et al., 2013; Jankowiak et al., 2022). An important observation may be that the predictions of perception-oriented models might be testable when information typicality/relevance (Bless et al., 1996), cognitive biases (Van Berkum et al., 2013), and general knowledge violations (Vissers et al., 2013; Jankowiak et al., 2022) are manipulated. Second, mood has been argued to regulate *attention*: positive and negative moods are, respectively, associated with global (i.e., top-down and broad) and narrow (i.e., bottom-up, local, and detail-oriented) attentional focus (e.g., Schwarz and Clore, 1983; Herz et al., 2020). These predictions are rather consistent with the reviewed behavioural (Bless et al., 1996; Rowe et al., 2006; *cf.* Sakaki et al., 2011) and electrophysiological evidence (Naranowicz et al., 2022b). It is noteworthy, however, that these studies drew conclusions about the breadth of attentional focus based on their findings from perceptual tasks (Bless et al., 1996; Rowe et al., 2006) or the pre-lexical stage of visual word processing (Naranowicz et al., 2022b), suggesting that research on semantic processing alone may not deepen our understanding of mood effects on attention to a great extent. Third, mood has also been hypothesised to affect *motivation*: a positive mood signals cognitive ease (i.e., effortless processing) and a need for maintenance of such a favourable state, whereas a negative mood marks cognitive difficulty (i.e., vigilant and effortful processing) and a need for one to improve their state of mind (e.g., Clark and Isen, 1982; Schwarz and Clore, 1983; Schwarz, 1990, 2002). While some researchers interested in the mood–semantics interactions have speculated that positive mood-driven facilitatory effects on semantic processing might be correlated with increased motivation (Van Berkum et al., 2013; Vissers et al., 2013), it appears that none of them have tested potential mood-dependent motivation effects in a systematic way. Fourth, mood has been anticipated to regulate *exploration tendencies*: positive and negative moods prompt exploratory or exploitatory behaviour, respectively (e.g., Zadra and Clore, 2011; Herz et al., 2020). Similarly to the motivation-oriented frameworks, this approach has not been addressed in research on the mood–semantics interactions in a systematic way. Still, Van Berkum et al. (2013) suggested that a negative mood may impair openness to exploratory processing, impeding heuristic anticipation.

Crucially, the available theoretical frameworks have not been oriented towards the role of mood in semantic processing, a notable exception being the ALC model offered by van Berkum et al. (2018; Van Berkum, 2019), which still only acknowledges a mediating role of the recipient’s mood in understanding messages from interlocutors. Observably, most behavioural and electrophysiological evidence has concentrated on how mood inhibits/impairs information retrieval from semantic memory and the relationships among concepts in it (e.g., Rowe et al., 2006; Storbeck and Clore, 2008; Chwilla et al., 2011; Naranowicz et al., 2022b). With accumulating evidence on mood effects on semantic memory organisation, future research could also concentrate on how to incorporate one’s mood state into theoretical models of semantic memory (see Kumar, 2021 for a review).

It is also noteworthy that, while most theoretical accounts somewhat complement one another, the SoM framework (Herz et al., 2020) seems to contradict earlier accounts in terms of its predictions about the mood–perception interactions. Specifically, in contrast to earlier theoretical frameworks (e.g., Schwarz and Clore, 1983; Bless, 2001; Bless and Fiedler, 2006), Herz et al. (2020) proposed that an increase in one’s mood is, among others, accompanied by increased reliance on sensory information (i.e., a broader SoM) whereas a decrease in mood with increased reliance on predictions (i.e., a narrower SoM). While the relationships between most dimensions in the SoM framework were hypothesised based on previous empirical work, Herz et al. (2020) did not actually offer much corroborative evidence to support such a mood–perception dependency. In fact, besides contradicting previous theoretical accounts, such a pattern does not seem to find much support in the discussed research on semantic processing, indicating that it is a positive and not negative that promotes reliance on previous knowledge and predictions (e.g., Chwilla et al., 2011; Van Berkum et al., 2013; Vissers et al., 2013). Moreover, moving beyond language research, increased reliance on pre-existing knowledge (e.g., cognitive biases) in a positive and not negative mood has also been observed in other domains (see Forgas, 2017 for a review). For instance, employing a shooter bias paradigm, Unkelbach et al. (2008) found that individuals in a positive mood may display increased aggressive tendencies towards Muslims (i.e., the *turban effect*) compared to those in a negative mood. Surprisingly, however, Herz’s et al. (2020) predictions about the other state of mind dimensions (i.e., attention, thought, and openness to experience) are still consistent with the earlier theoretical accounts – they proposed that a positive mood may be associated with a global attentional focus, broader associative thinking, and exploratory tendencies whereas a negative mood with a local attentional focus, narrow accommodative thinking, and exploitatory tendencies (e.g., Schwarz and Clore, 1983; Bless, 2001; Zadra and Clore, 2011). Therefore, though the SoM framework (Herz et al., 2020) offers a comprehensive view on the role of mood in one’s overall state of mind, its predictions about the mood–perception relationship does not seem to be sufficiently supported by previous research and should be interpretated with caution.

### Methodological considerations

To test predictions about mood–language interactions, researchers have elicited positive and negative mood states using a range of MIPs. Affectively evocative film clips appear to be the option of choice in psycholinguistic research due to their high potency. Experimentally induced mood fluctuations have been traditionally measured using self-reports, which coincides with the dimensional (e.g., Russell, 1980) and distinct-state (e.g., Izard, 1993) approaches to affective constructs. Although easy to administer, such measures are subject to a number of measurement issues that may question their reliability, such as the social reliability bias, obeying demand characteristics, or variations in participants’ intrapersonal skills.

Arguably, a critical methodological issue concerning experimental mood elicitation is the selection of an effective measure of mood change. Ekkekakis (2013) argued that a measure of an affective construct of interest (i.e., core affect, mood, or emotion) should be consistent with a theoretical framework upon which the measure was built. For instance, adopting the dimensional approach to mood would necessitate using bipolar mood valence (i.e., positive––negative) and arousal (calm––excited) scales to measure experimentally induced mood changes. While such a consistency-driven perspective is reasonable, it could also be justifiable, if not recommended, to adopt a more practical perspective: employing a broader spectrum of mood measurements in research involving positive and negative mood elicitation. The revised literature suggests that, when implemented through standardised procedures, elicitation of positive and negative moods typically affects participants’ mood ratings in a predictable manner. Specifically, when a bipolar mood valence scale (i.e., positive––negative) is adopted, it is reasonable to expect an increase/no change in mood ratings post relative to pre mood induction in the positive mood condition and their decrease in the negative mood condition (e.g., Wang et al., 2016). An analogous pattern expected for two unipolar scales is a negative correlation between mood ratings in both mood conditions: higher mood ratings post mood induction on the positive mood/happiness scale are typically accompanied by lower mood ratings on the negative mood/sadness scale in the positive mood condition, with the reversed pattern in the negative mood condition (Joseph et al., 2020). An additional use of such unipolar scales would help researchers better understand the relationship between positive and negative moods elicited *via* MIPs. For instance, it is probable that decreased mood ratings on a bipolar scale are reflective of a decreased positive mood without increasing a negative mood (see Joseph et al., 2020), which could significantly change the interpretation of observed mood effects on cognitive processes. Furthermore, even when mood induction aims to elicit general positive and negative moods, individual mood-inducing stimuli could evoke discrete affective states of varying intensity due to their individual characteristics or participants’ personal associations. Therefore, it seems also reasonable to supplement bipolar and unipolar scales with a mood-related questionnaire targeting discrete positive and negative emotions, which may again help mood researchers better understand the complexity of the affective states evoked by their mood manipulation (e.g., Naranowicz et al., 2022a,b).

Nevertheless, given the elusive nature of our affective states, it is difficult, if at all possible, to objectively and accurately measure participants’ current mood. Mood researchers could also benefit greatly from employment of various physiological measures (e.g., heart rate variability or skin conductance responses) to measure participants’ reactivity to mood-inducing stimuli in a more objective fashion (e.g., Engelbregt et al., 2022; Sterenberg Mahon and Roth, 2022). For instance, electrodermal activity measures (e.g., skin conductance responses) have been used as a physiological marker of changes in the sympathetic nervous system reflecting one’s emotional arousal (see Behnke et al., 2022 for a review).

### Behavioural and electrophysiological evidence

Behavioural research has pointed to differences in how positive and negative moods affect semantic processes, concentrating mostly on semantic memory organisation, reliance on pre-existing knowledge, and attentional focus. Specifically, a positive mood has been observed to facilitate the spread of activation to close associates (Hänze and Hesse, 1993) and/or remote associates (Bolte et al., 2003) in semantic memory. Such a favourable mood state has also been associated with a greater breadth of attentional selection (i.e., a global attentional focus; Rowe et al., 2006) as well as reliance on general knowledge structures (i.e., heuristics) due to increased cognitive ease (i.e., effortless processing; Bless et al., 1996). In contrast, a negative mood has also been linked to dampened activation of semantic associations in semantic memory (Storbeck and Clore, 2008; Sakaki et al., 2011), which might actually be limited to close associates (Bolte et al., 2003), as well as decreased breadth of attentional selection (i.e., a local attentional focus; Rowe et al., 2006). A negative mood has also been linked to impeded sentence comprehension particularly in older relative to younger adults (Liu, 2021). On the other hand, a negative mood has also been found to facilitate responses to systematic stimuli requiring controlled processing (Hesse and Spies, 1996) and result in greater attention to detail and an accommodative processing style (Matovic et al., 2014).

Similarly to behavioural investigations, electrophysiological research has also pointed to marked between-mood differences in semantic processing, offering explanations based on attention, motivation, and processing strategies. A positive mood has been linked to facilitated lexicosemantic access to both distantly related concepts (i.e., between-category violations; Federmeier et al., 2001) and those belonging to the same semantic category (i.e., within-category violations; Pinheiro et al., 2013). It has also been associated with the activation of a global, heuristics-based processing style during lexicosemantic access (Chwilla et al., 2011) and semantic re-analysis (Vissers et al., 2013). It is noteworthy that this effect may be limited to bilinguals’ native language only (Naranowicz et al., 2022b) and observed mostly in females rather than males (Federmeier et al., 2001; see also Naranowicz et al., 2022a). Others have also suggested that a positive relative to negative mood may lead to increased motivation (Vissers et al., 2013) along with allocation of attentional resources to the most relevant contextual information (Vissers et al., 2013; Wang et al., 2016). In contrast, being in a negative mood may result in increased sensitivity to contextual information due to the activation of detail-oriented processing (Pinheiro et al., 2013), especially during semantic re-analysis (Chwilla et al., 2011; Vissers et al., 2013; Wang et al., 2016), inhibition of associative retrieval from long-term memory (Van Berkum et al., 2013), and increased working memory demands (Vissers et al., 2013).

Together, the discussed behavioural and electrophysiological evidence has demonstrated that positive and negative moods differently affect semantic processes, which is consistent with a common finding that these two mood states promote different cognitive strategies (i.e., mood-dependent processing styles; see Forgas, 2017 for a review). However, the reviewed literature has also revealed a number of discrepancies in empirical findings, which may somewhat distort this clear picture.

First, while a positive mood has been observed to exert an overall facilitatory effect on semantic processes, it remains unclear if closely and remotely associates are affected by it to the same degree. Specifically, Hänze and Hesse (1993) observed a facilitative impact of a positive mood on spreading activation to close yet not remote associates, whereas Bolte et al. (2003) observed such a pattern for remote associates. Such a discrepancy could be accounted for by methodological differences: Hänze and Hesse (1993) employed a semantic priming paradigm, relying on RTs in an LDT, and Bolte et al. (2003) employed a remote association paradigm, relying on the accuracy of intuitive coherence judgements in a remote association task. Interestingly, a similar discrepancy can also be observed in electrophysiological research. Federmeier et al. (2001) found a facilitatory effect of a positive mood on lexicosemantic access to remote associates (i.e., a reduced N400 response to BCVs in a positive relative to neutral mood), whereas Pinheiro et al. (2013) to close associates (i.e., a reduced N400 response to WCVs in a positive relative to neutral mood). Pinheiro et al. (2013) suggested that the differential mood effects observed in the two studies may have been driven by stimuli (different items), MIP (i.e., presentation of many emotional pictures at once vs. one picture before each sentence), gender (females vs. males), and task instructions (i.e., passive reading vs. an SDT). It is also noteworthy that the sample sizes in both studies were rather limited: Federmeier et al. (2001) recruited 11 female participants and Pinheiro et al. (2013) 15 male participants. Hence, future research on mood–language interactions could benefit greatly from a replication-oriented approach. The studies reviewed above have employed the whole spectrum of semantically oriented tasks as well as mood-inducing and linguistic stimuli. On the one hand, this approach is advantageous seeing that each study broadens our knowledge about mood–language interactions. On the other hand, numerous procedural differences make it impossible to draw valid conclusions, including those about mood effects on remote and close associates. Whenever possible, it would be beneficial to undertake conceptual replications (i.e., changing only one dimension).

Second, another inconsistency observed in electrophysiological research concerns the mood-driven N400 amplitude changes. While many researchers have observed facilitatory effects of a positive mood on lexicosemantic access, as marked by the N400 amplitude changes (Federmeier et al., 2001; Chwilla et al., 2011; Pinheiro et al., 2013; Wang et al., 2016; Naranowicz et al., 2022b), others have actually failed to observe any mood-driven modulations in the N400 time frame (Jiménez-Ortega et al., 2012; Vissers et al., 2013; Goertz et al., 2017; Ogawa and Nittono, 2019b; Jankowiak et al., 2022). Such a null mood effect may be linked to the lexicosemantic mechanisms of interest (i.e., semantic plausibility vs. expectancy). For instance, both Vissers et al. (2013) and Jankowiak et al. (2022) built the semantically implausible sentences based on general world knowledge violations (i.e., unexpected and completely implausible sentences), whereas others have mostly employed the semantic anomalies based on expectancy (i.e., unexpected yet not entirely implausible sentences; e.g., Federmeier et al., 2001; Chwilla et al., 2011; Naranowicz et al., 2022b). Another reason for no mood effect on lexicosemantic access may be related to the use of weak/ineffective MIP. For instance, Jiménez-Ortega et al. (2012) found no mood effects in both the N400 and LPC time frames, concluding that the employed mood-inducing short written stories (i.e., four-sentence paragraphs) could have been of insufficient power to elicit significant mood effects. Using the experimental paradigms indirectly related to lexicosemantic processes may also be another reason for finding no mood effects in the N400 time window (Goertz et al., 2017; Ogawa and Nittono, 2019b). For instance, Ogawa and Nittono (2019b) explored how positive and negative moods affect subjective imaginability ratings and found no mood effects on the N400 and N700 components, explaining that such a null effect may be linked to the employment of a rating task instead of a binary decision–based task or decontextualized words instead of the words embedded in sentential contexts.

Third, previous research on the mood–language interactions has also produced somewhat inconsistent results regarding the breadth of attentional focus. Previous studies have pointed to increased breadth of attentional focus in a positive mood, which can also be narrowed in a negative mood (see Moriya and Nittono, 2011 for a review). Such a pattern is consistent with previous theoretical models (e.g., Schwarz and Clore, 1983; Zadra and Clore, 2011; Herz et al., 2020), and it has also been observed in the reviewed literature (Bless et al., 1996; Rowe et al., 2006; Naranowicz et al., 2022b). For instance, Rowe et al. (2006) observed slower RTs to incompatibility trials (i.e., strings of letters with one different letter) in a positive relative to neutral and negative mood in a Flanker task. They concluded that a positive mood may impair the selective visuospatial attention as a result of eased inhibitory control and, consequently, a broader attentional focus. Similarly, Naranowicz et al. (2022b) found a larger P1 (i.e., a marker of early sensory processing modulated by attention) response to words in a positive compared to negative mood, also associating such an effect with broadened attentional focus in a positive mood (see Moriya and Nittono, 2011 for corroborative evidence from a Flanker task). In contrast, Sakaki et al. (2011) found slower RTs to word pairs in an SCT, with no between-mood difference in perceptual tasks (i.e., judging the colour/shade of the first letter), concluding that a negative mood impedes semantic processing to a greater extent than perceptual processing. Yet, one could argue that Sakaki et al. (2011) study may not be suitable for drawing such conclusions, as the predictions about mood-driven attentional focus are typically tested using global–local visual processing paradigms (Moriya and Nittono, 2011). Moreover, Sakaki et al. (2011) also employed longer SOA of 1,300 ms, promoting controlled rather than attentional effects. Therefore, it appears that a negative mood does not necessary impairs semantic processing to a greater extent than perceptual processing, as suggested by Sakaki et al. (2011), and more research is needed to corroborate this finding.

Finally, another unresolved question is whether a negative mood sensitises us to contextual information. Besides pointing to an inhibitory effect of a negative mood on lexicosemantic access (i.e., impaired sensitivity to the relationship between concepts in semantic memory), Pinheiro et al. (2013) also suggested that a negative mood may lead to narrowed context-specific predictions, possibly being indicative of negative mood–driven selective attention. In contrast, Wang et al. (2016) suggested that a positive mood may promote selective attention to the most relevant contextual information, whereas a negative mood may promote non-selective attention to all semantic relations. Based on such findings, one could tentatively conclude that a negative mood may promote selective attention when expected semantic information is processed (as in Pinheiro et al., 2013) and non-selective attention in the presence of semantic violations (as in Wang et al., 2016). Yet, bearing in mind that the two findings have not yet been replicated by other ERP studies as well as numerous methodological differences between the two studies, more research is needed to answer the question whether and how a negative mood promotes (non-)selective contextual sensitivity.

Regarding the methodological considerations, behavioural and electrophysiological research has targeted mood–semantic processing interactions and mostly reached comparable conclusions, but these two bodies of research have focused on distinct aspects of semantic processing and employed dissimilar experimental paradigms. Namely, behavioural research has mostly investigated mood effects on semantic memory structure and spread of activation as indexed by RTs, response accuracy, and information recall, manipulating semantic relatedness, congruity, and categories mostly at a word level (e.g., Storbeck and Clore, 2008). In contrast, electrophysiological research has mostly concentrated on mood effects on lexicosemantic access and semantic re-evaluation (i.e., two consecutive meaning-related stages of visual word processing), as indexed by N400 and LPC modulations, manipulating semantic congruity and plausibility primarily at sentence and discourse levels (e.g., Chwilla et al., 2011). Behavioural research on mood–language interactions appears to slowly transition to electrophysiological research. This seems to be a natural direction since behavioural measures have known limitations due to response latencies and accuracy reflecting the end product of the whole meaning-driven decision-making process (Liu, 2021), whereas ERP components can index online brain activity changes throughout the full time-window of processing. To test the reliability and validity of the behavioural findings and to provide a fresh perspective on them, future work could adopt, for instance, the semantic priming (e.g., Sakaki et al., 2011) or remote association (e.g., Rowe et al., 2006) paradigms in ERP experiments.

It is also noteworthy that there have emerged two approaches to interpreting the N400 modulations by mood. A linear decline in the N400 amplitudes is typically interpreted as indicative of enhanced lexicosemantic access, which translates into facilitated retrieval of word meaning from long-term memory due to increased cognitive ease and the activation of more predictive mechanisms (e.g., Kutas and Hillyard, 1980). Consequently, most researchers have interpreted a decrease in N400 amplitudes/a smaller N400 effect in a positive compared to neutral and/or negative mood as indicative of positive mood–driven facilitation of lexicosemantic access (e.g., Federmeier et al., 2001; Pinheiro et al., 2013; Naranowicz et al., 2022b). Chwilla et al. (2011), however, observed a broadly and bilaterally distributed N400 effect in a positive mood, with its significant reduction to the right hemisphere and the left occipital and temporal sites in a negative mood. Consequently, Chwilla et al. (2011) proposed an alternative approach, whereby mood-dependent effects on lexicosemantic access are reflected in the N400 effect distribution instead of the N400 amplitude changes.

Speculatively, a potential cause of the frequently reported dichotomous mood-dependent processing styles may also be rooted in the “file drawer” phenomenon – a common scientific practice of not publishing research producing null results (Mervis, 2014). There may thus be evidence pointing to similarities between positive and negative mood effects on semantic processing which has never been published. Ogawa and Nittono (2019a,b), for instance, looked at positive and negative moods influences on word imagery processing, as indexed by N400 and N170 components as well as RTs. With the exception of one main effect of mood (i.e., a larger N400 response in a positive than negative mood), both studies failed to reveal any differential effect of mood, despite adopting standardised experimental procedures for mood research and sufficient sample size. Ogawa and Nittono (2019a,b) research represents desirable practice that may help the scientific community counteract the current *replication crisis* (see Shrout and Rodgers, 2018, for a review) and potentially shed a fresh light on research on mood–language interaction: the authors made their unpublished manuscript available online, pre-registered their follow-up study, calculated their sample size in advance, and shared their primary data.

### Other future research directions

One of the outstanding questions in mood research concerns the possibility of gender differences in mood–language interactions. Federmeier et al. (2001) was the first to report a stronger facilitatory effect of a positive mood on semantic processing in females than males, which was later corroborated in a recent behavioural study (Naranowicz et al., 2022a). Such a female advantage in a positive mood may possibly be explained by greater sensitivity to emotions (e.g., Goldstein et al., 2001) or increased physiological reactivity to affective stimuli (e.g., Bianchin and Angrilli, 2012; Naranowicz et al., 2022a). Moreover, given that females are stereotypically perceived as more emotional than males (e.g., Fischer, 1993), they might even be more susceptible to mood induction due to a social desirability bias (i.e., they might believe that this is a socially expected behaviour from them). It is noteworthy that many studies discussed above have in fact concentrated on female participants only (e.g., Chwilla et al., 2011; Van Berkum et al., 2013; Vissers et al., 2013; Wang et al., 2016; Jankowiak et al., 2022; Naranowicz et al., 2022b), indicating that mood and affective research itself may be somewhat biased towards testing or reporting data from the female population only. More attention should therefore be devoted to cross-sex comparisons to better understand the potential mood–gender interactions in linguistic research and to make observed findings more generalisable. Also, future research could benefit from approaching gender as a non-binary social construct, especially given that non-binary persons constitute a marginalised and under-researched population (e.g., Richards et al., 2016).

Another outstanding question in the mood–language literature is the practical implications of previous empirical evidence, particularly in the case of psychotherapy, interpersonal communication, education, mediation, and negotiation. For instance, one could expect that our current mood may influence communicative interactions. Specifically, while being in a good mood may potentially improve overall comprehension of the messages communicated to us, it may also make us think more stereotypically, leading to potential misunderstandings. Then, being in a negative mood may excessively direct our attention to details during communicative encounters, making us miss a bigger picture. Another interesting example is psychotherapy. Though the reviewed literature did not regard clinical populations, one could expect individuals with depression to benefit greatly from talking about their emotions in their L2 when they are unable to communicate them freely in their L1 (Naranowicz et al., 2022b).

## Conclusion

The discussed theoretical frameworks have offered rather consistent predictions about how mood affects perception, attention, motivation, and exploration tendencies. The reviewed behavioural and electrophysiological research has provided the greatest empirical support for the perception-oriented accounts.

The present paper reviewed research on positive and negative mood effects on semantic processing so as to offer some future research directions. First, while the discussed perception- and attention-oriented theoretical frameworks have found empirical support in research on mood and semantic processing (e.g., Jankowiak et al., 2022; Naranowicz et al., 2022b), there is a need for incorporating mood into semantic memory-oriented models. Second, it would be reasonable to employ a broader spectrum of mood measures when eliciting positive and negative moods, which would help understand the dynamics of participants’ affective states. Third, because of the discrepancies in the observed findings, more scholarly attention should be devoted to the questions of how mood affects close and remote associates in semantic memory (e.g., Federmeier et al., 2001; Pinheiro et al., 2013), lexicosemantic access as indexed by the N400 amplitude changes (e.g., Chwilla et al., 2011; Ogawa and Nittono, 2019b), attentional breadth (e.g., Rowe et al., 2006; Sakaki et al., 2011), and (non-)selective contextual sensitivity (e.g., Pinheiro et al., 2013; Wang et al., 2016). Fourth, a replication-oriented approach could be advantageous to research on mood–semantic processing interactions in order to account for some unanticipated results. Finally, the frequently reported dichotomous mood-dependent processing styles could potentially result from a publication bias, and good research practices such as pre-registration could help researchers identify potential similarities between positive and negative moods effects on semantic processing.

## Author contributions

MN contributed to conceptualisation as well as manuscript writing and revision.

## Funding

This work was funded by the National Science Centre, Poland (Grant No 2018/31/N/HS2/00120) awarded to MN.

## Conflict of interest

The author declares that the research was conducted in the absence of any commercial or financial relationships that could be construed as a potential conflict of interest.

## Publisher’s note

All claims expressed in this article are solely those of the authors and do not necessarily represent those of their affiliated organizations, or those of the publisher, the editors and the reviewers. Any product that may be evaluated in this article, or claim that may be made by its manufacturer, is not guaranteed or endorsed by the publisher.

## References

[ref1] Bar M. (2021). From objects to unified minds. Curr. Dir. Psychol. Sci. 30, 129–137. doi: 10.1177/0963721420984403, PMID: 32738590

[ref2] Baumeister R. F. Bratslavsky E. Finkenauer C. Vohs K. D. (2001). Bad is stronger than good. Rev. Gen. Psychol. 5, 323–370. doi: 10.1037/1089-2680.5.4.323, PMID: 36274158

[ref3] Behnke M. Kreibig S. D. Kaczmarek L. D. Assink M. Gross J. J. (2022). Autonomic nervous system activity during positive emotions: a meta-analytic review. Emot. Rev. 14, 132–160. doi: 10.1177/17540739211073084

[ref4] Beukeboom C. J. Semin G. R. (2006). How mood turns on language. J. Exp. Soc. Psychol. 42, 553–566. doi: 10.1016/j.jesp.2005.09.005, PMID: 36138773

[ref5] Bianchin M. Angrilli A. (2012). Gender differences in emotional responses: a psychophysiological study. Physiol. Behav. 105, 925–932. doi: 10.1016/j.physbeh.2011.10.031, PMID: 22108508

[ref6] Bless H. (2001). “Mood and the use of general knowledge structures” in Theories of mood and cognition: A user’s guidebook. eds. Martin L. L. Clore G. L. (Mahwah, NJ, US: Lawrence Erlbaum Associates Publishers), 9–26.

[ref7] Bless H. Fiedler K. (2006). “Mood and the regulation of information processing and behavior” in Affect in social thinking and behavior. ed. Forgas J. P. (New York, NY: Psychology Press), 65–84.

[ref8] Bless H. Schwarz N. Clore G. L. Golisano V. Rabe C. Wölk M. (1996). Mood and the use of scripts: does a happy mood really lead to mindlessness? J. Pers. Soc. Psychol. 71, 665–679. doi: 10.1037/0022-3514.71.4.665, PMID: 8888596

[ref9] Bohn-Gettler C. M. Rapp D. N. (2011). Depending on my mood: mood-driven influences on text comprehension. J. Educ. Psychol. 103, 562–577. doi: 10.1037/a0023458, PMID: 21927504 PMC3172144

[ref10] Bolte A. Goschke T. Kuhl J. (2003). Emotion and intuition: effects of positive and negative mood on implicit judgments of semantic coherence. Psychol. Sci. 14, 416–421. doi: 10.1111/1467-9280.01456, PMID: 12930470

[ref12] Braun N. Goudbeek M. Krahmer E. (2019). Language and emotion – a foosball study: the influence of affective state on language production in a competitive setting. PLoS One 14:e0217419. doi: 10.1371/journal.pone.0217419, PMID: 31125388 PMC6534325

[ref13] Brehm J. W. Miron A. M. (2006). Can the simultaneous experience of opposing emotions really occur? Motiv. Emot. 30, 13–30. doi: 10.1007/s11031-006-9007-z

[ref14] Brouwer H. Fitz H. Hoeks J. (2012). Getting real about semantic illusions: rethinking the functional role of the P600 in language comprehension. Brain Res. 1446, 127–143. doi: 10.1016/j.brainres.2012.01.055, PMID: 22361114

[ref15] Carvalho S. Leite J. Galdo-Álvarez S. Gonçalves Ó. F. (2012). The emotional Movie database (EMDB): a self-report and psychophysiological study. Appl. Psychophysiol. Biofeedback 37, 279–294. doi: 10.1007/s10484-012-9201-6, PMID: 22767079

[ref16] Chung G. Tucker D. M. West P. Potts G. F. Liotti M. Luu P.. (1996). Emotional expectancy: brain electrical activity associated with an emotional bias in interpreting life events. Psychophysiology 33, 218–233. doi: 10.1111/j.1469-8986.1996.tb00419.x, PMID: 8936391

[ref17] Chwilla D. J. Virgillito D. Vissers C. T. (2011). The relationship of language and emotion: N400 support for an embodied view of language comprehension. J. Cogn. Neurosci. 23, 2400–2414. doi: 10.1162/jocn.2010.21578, PMID: 20849229

[ref18] Clark M. S. Isen A. M. (1982). Toward understanding the relationship between feeling states and social behaviour. Cognitive Soc. Psychol. 73–108.

[ref19] Clore G. L. Gasper K. Garvin E. (2001). “Affect as information” in Handbook of affect and social cognition. ed. Forgas J. P. (Mahwah, NJ, US: Lawrence Erlbaum Associates Publishers), 121–144.

[ref20] Clore G. L. Storbeck J. (2006). “Affect as information about liking, efficacy, and importance” in Affect in social thinking and behavior. ed. Forgas J. P. (New York, NY, US: Psychology Press), 123–141.

[ref22] Dwivedi V. D. Selvanayagam J. (2021). Effects of dispositional affect on the N400: language processing and socially situated context. Front. Psychol. 12:566894. doi: 10.3389/fpsyg.2021.566894, PMID: 33868066 PMC8044537

[ref23] Egidi G. Nusbaum H. C. (2012). Emotional language processing: how mood affects integration processes during discourse comprehension. Brain Lang. 122, 199–210. doi: 10.1016/j.bandl.2011.12.008, PMID: 22325258

[ref24] Ekkekakis P. (2013). The measurement of affect, mood, and emotion: A guide for health-Behavioral research. Cambridge: Cambridge University Press.

[ref25] Ekman P. (1992). An argument for basic emotions. Cognit. Emot. 6, 169–200. doi: 10.1080/02699939208411068

[ref26] Elman P. (1994). The nature of emotion: Fundamental questions. New York, NY, US: Oxford University Press.

[ref27] Engelbregt H. J. Brinkman K. van Geest C. C. E. Irrmischer M. Deijen J. B. (2022). The effects of autonomous sensory meridian response (ASMR) on mood, attention, heart rate, skin conductance and EEG in healthy young adults. Exp. Brain Res. 240, 1727–1742. doi: 10.1007/s00221-022-06377-9, PMID: 35511270 PMC9142458

[ref28] Fakhr Hosseini M. Jeon M. (2017). “Chapter 10 - affect/emotion induction methods” in Emotions and affect in human factors and human-computer interaction. ed. Jeon M. (San Diego: Academic Press), 235–253.

[ref29] Federmeier K. D. Kirson D. A. Moreno E. M. Kutas M. (2001). Effects of transient, mild mood states on semantic memory organization and use: an event-related potential investigation in humans. Neurosci. Lett. 305, 149–152. doi: 10.1016/S0304-3940(01)01843-2, PMID: 11403927

[ref30] Fernández Megías C. Pascual Mateos J. C. Soler Ribaudi J. Fernández-Abascal E. G. (2011). Spanish validation of an emotion-eliciting set of films. Psicothema 23, 778–785. PMID: 22047873

[ref31] Fernández-Aguilar L. Ricarte J. Ros L. Latorre J. M. (2019). Emotional differences in young and older adults: films as mood induction procedure. Front. Psychol. 9:1110. doi: 10.3389/fpsyg.2018.01110 30018584 PMC6037940

[ref32] Fischer A. H. (1993). Sex differences in emotionality: fact or stereotype? Feminism and Psychology, 3, 303–318. doi: 10.1177/0959353593033002

[ref33] Forgas J. P. (1995). Mood and judgment: the affect infusion model (AIM). Psychol. Bull. 117, 39–66. doi: 10.1037/0033-2909.117.1.39, PMID: 7870863

[ref34] Forgas J. P. (1999). Feeling and speaking: mood effects on verbal communication strategies. Personal. Soc. Psychol. Bull. 25, 850–863. doi: 10.1177/0146167299025007007

[ref35] Forgas J. (2002). Feeling and doing: affective influences on interpersonal behavior. Psychol Inquiry 13, 1–28. doi: 10.1207/S15327965PLI1301_01

[ref36] Forgas J. P. (2017). “Chapter 3 - mood effects on cognition: affective influences on the content and process of information processing and behavior” in Emotions and affect in human factors and human-computer interaction. ed. Jeon M. (San Diego: Academic Press), 89–122.

[ref37] Forgas J. P. Matovic D. (2020). Mood effects on humor production: positive mood improves the verbal ability to be funny. J. Lang. Soc. Psychol. 39, 701–715. doi: 10.1177/0261927X20917994

[ref38] Friedman D. Johnson R. Jr. (2000). Event-related potential (ERP) studies of memory encoding and retrieval: a selective review. Microsc. Res. Tech. 51, 6–28. doi: 10.1002/1097-0029(20001001)51:1<6::AID-JEMT2>3.0.CO;2-R, PMID: 11002349

[ref39] Frijda N. H. (2009). “Mood” in The Oxford companion to emotion and the affective sciences. eds. Sander D. Scherer K. R. (New York: Oxford University Press), 258–259.

[ref40] Goertz R. I. Vissers C. T. W. M. Verhees M. W. F. T. Chwilla D. J. (2017). Effects of emotional state on processing conceptual mismatches: ERPs in a picture-sentence matching task. JBBS 7, 557–584. doi: 10.4236/jbbs.2017.712040

[ref41] Goldstein J. M. Seidman L. J. Horton N. J. Makris N. Kennedy D. N. Caviness V. S.. (2001). Normal sexual dimorphism of the adult human brain assessed by in vivo magnetic resonance imaging. Cereb. Cortex 11, 490–497. doi: 10.1093/cercor/11.6.490, PMID: 11375910

[ref42] Gray E. K. Watson D. (2007). “Assessing positive and negative affect via self-report” in Handbook of emotion elicitation and assessment series in affective science. eds. Coan J. A. Allen J. J. B. (New York, NY, US: Oxford University Press), 171–183.

[ref43] Hagoort P. Brown C. Groothusen J. (1993). The syntactic positive shift (sps) as an erp measure of syntactic processing. Language and Cognitive Processes 8, 439–483. doi: 10.1080/01690969308407585

[ref44] Hänze M. Hesse F. W. (1993). Emotional influences on semantic priming. Cognit. Emot. 7, 195–205. doi: 10.1080/02699939308409184 27102737

[ref45] Herz N. Baror S. Bar M. (2020). Overarching states of mind. Trends Cogn. Sci. 24, 184–199. doi: 10.1016/j.tics.2019.12.015, PMID: 32059121

[ref46] Hesse F. W. Spies K. (1996). Effects of negative mood on performance: reduced capacity or changed processing strategy? Eur. J. Soc. Psychol. 26, 163–168. doi: 10.1002/(SICI)1099-0992(199601)26:1<163::AID-EJSP749>3.0.CO;2-W, PMID: 36216360

[ref47] Hinojosa J. A. Fernández-Folgueiras U. Albert J. Santaniello G. Pozo M. A. Capilla A. (2017). Negative induced mood influences word production: an event-related potentials study with a covert picture naming task. Neuropsychologia 95, 227–239. doi: 10.1016/j.neuropsychologia.2016.12.025, PMID: 28025016

[ref49] Isen A. M. Johnson M. M. S. Mertz E. Robinson G. F. (1985). The influence of positive affect on the unusualness of word associations. J. Pers. Soc. Psychol. 48, 1413–1426. doi: 10.1037/0022-3514.48.6.1413, PMID: 4020605

[ref50] Izard C. E. (1993). “Organizational and motivational functions of discrete emotions” in Handbook of emotions. eds. Lewis M. Haviland J. M. (New York, NY, US: The Guilford Press), 631–641.

[ref51] Jankowiak K. Naranowicz M. Thierry G. (2022). Positive and negative moods differently affect creative meaning processing in both the native and non-native language. Brain Lang. 235:105188. doi: 10.1016/j.bandl.2022.105188, PMID: 36242817

[ref52] Jiménez-Ortega L. Martín-Loeches M. Casado P. Sel A. Fondevila S. de Tejada P. H.. (2012). How the emotional content of discourse affects language comprehension. PLoS One 7:e33718. doi: 10.1371/journal.pone.0033718, PMID: 22479432 PMC3315581

[ref53] Joormann J. Stanton C. H. (2016). Examining emotion regulation in depression: a review and future directions. Behav. Res. Ther. 86, 35–49. doi: 10.1016/j.brat.2016.07.007, PMID: 27492851

[ref54] Joseph D. L. Chan M. Y. Heintzelman S. J. Tay L. Diener E. Scotney V. S. (2020). The manipulation of affect: a meta-analysis of affect induction procedures. Psychol. Bull. 146, 355–375. doi: 10.1037/bul0000224, PMID: 31971408

[ref55] Kharkhurin A. V. Altarriba J. (2016). The effect of mood induction and language of testing on bilingual creativity. Bilingualism 19, 1079–1094. doi: 10.1017/S1366728915000528

[ref56] Kiefer M. Schuch S. Schenck W. Fiedler K. (2007). Mood states modulate activity in semantic brain areas during emotional word encoding. Cereb. Cortex 17, 1516–1530. doi: 10.1093/cercor/bhl062, PMID: 16926240

[ref57] Kissler J. Bromberek-Dyzman K. (2021). Mood induction differently affects early neural correlates of evaluative word processing in L1 and L2. Front. Psychol. 11:588902. doi: 10.3389/fpsyg.2020.588902, PMID: 33510673 PMC7835133

[ref58] Koch A. S. Forgas J. P. Matovic D. (2013). Can negative mood improve your conversation? Affective influences on conforming to Grice’s communication norms. Eur. J. Soc. Psychol. 43, 326–334. doi: 10.1002/ejsp.1950

[ref59] Kumar A. A. (2021). Semantic memory: a review of methods, models, and current challenges. Psychon. Bull. Rev. 28, 40–80. doi: 10.3758/s13423-020-01792-x, PMID: 32885404

[ref60] Kuperberg G. R. Sitnikova T. Caplan D. Holcomb P. J. (2003). Electrophysiological distinctions in processing conceptual relationships within simple sentences. Cogn. Brain Res. 17, 117–129. doi: 10.1016/S0926-6410(03)00086-7, PMID: 12763198

[ref61] Kutas M. Hillyard S. A. (1980). Event-related brain potentials to semantically inappropriate and surprisingly large words. Biol. Psychol. 11, 99–116. doi: 10.1016/0301-0511(80)90046-0, PMID: 7272388

[ref62] Kutas M. Hillyard S. A. (1984). Brain potentials during reading reflect word expectancy and semantic association. Nature 307, 161–163. doi: 10.1038/307161a0, PMID: 6690995

[ref63] Lazarus R. S. (1991). Emotion and adaptation. New York, NY: Oxford University Press.

[ref64] Lench H. C. Flores S. A. Bench S. W. (2011). Discrete emotions predict changes in cognition, judgment, experience, behavior, and physiology: a meta-analysis of experimental emotion elicitations. Psychol. Bull. 137, 834–855. doi: 10.1037/a0024244, PMID: 21766999

[ref01] Liu X. (2021). The effect of mood on predictive sentence processing by Older Adults. SAGE Open 11:215824402110156. doi: 10.1177/21582440211015683

[ref65] Liu X. Xu X. Wang H. (2018). The effect of emotion on morphosyntactic learning in foreign language learners. PLoS One 13:e0207592. doi: 10.1371/journal.pone.0207592, PMID: 30475844 PMC6261014

[ref66] Lovibond P. F. Lovibond S. H. (1995). The structure of negative emotional states: comparison of the depression anxiety stress scales (DASS) with the Beck depression and anxiety inventories. Behav. Res. Ther. 33, 335–343. doi: 10.1016/0005-7967(94)00075-U, PMID: 7726811

[ref67] Maffei A. Angrilli A. (2019). E-MOVIE - experimental MOVies for induction of emotions in neuroscience: an innovative film database with normative data and sex differences. PLoS One 14:e0223124. doi: 10.1371/journal.pone.0223124, PMID: 31581254 PMC6776321

[ref68] Matovic D. Forgas J. P. (2018). The answer is in the question? Mood effects on processing verbal information and impression formation. J. Lang. Soc. Psychol. 37, 578–590. doi: 10.1177/0261927X18764544

[ref69] Matovic D. Koch A. S. Forgas J. P. (2014). Can negative mood improve language understanding? Affective influences on the ability to detect ambiguous communication. J. Exp. Soc. Psychol. 52, 44–49. doi: 10.1016/j.jesp.2013.12.003

[ref70] McNair D. M. Lorr M. Droppleman L. F. (1971). Manual for the profile of mood states. United States: Educational and Industrial Testing Services.

[ref71] Mervis J. (2014). Why null results rarely see the light of day. Science 345:992. doi: 10.1126/science.345.6200.992, PMID: 25170131

[ref72] Mills C. Wu J. D’Mello S. (2019). Being sad is not always bad: the influence of affect on expository text comprehension. Discourse Process. 56, 99–116. doi: 10.1080/0163853X.2017.1381059

[ref74] Moreno E. Kutas M. (2005). Processing semantic anomalies in two languages: an electrophysiological exploration in both languages of Spanish-English bilinguals. Brain Res. Cogn. Brain Res. 22, 205–220. doi: 10.1016/j.cogbrainres.2004.08.010, PMID: 15653294

[ref75] Moriya H. Nittono H. (2011). Effect of mood states on the breadth of spatial attentional focus: an event-related potential study. Neuropsychologia 49, 1162–1170. doi: 10.1016/j.neuropsychologia.2011.02.036, PMID: 21352834

[ref76] Morris W. N. (1992). “A functional analysis of the role of mood in affective systems” in Emotion review of personality and social psychology, vol. 13. ed. Clark M. S. (Thousand Oaks, CA, US: Sage Publications, Inc), 256–293.

[ref77] Naranowicz M. Jankowiak K. Bromberek-Dyzman K. (2022a). Mood and gender effects in emotional word processing in unbalanced bilinguals. Int. J. Biling. 13670069221075646:136700692210756. doi: 10.1177/13670069221075646

[ref78] Naranowicz M. Jankowiak K. Kakuba P. Bromberek-Dyzman K. Thierry G. (2022b). In a bilingual mood: mood affects Lexicosemantic processing differently in native and non-native languages. Brain Sci. 12:316. doi: 10.3390/brainsci12030316, PMID: 35326272 PMC8945979

[ref79] Nielsen L. Kaszniak A. W. (2007). “Conceptual, theoretical, and methodological issues in inferring subjective emotion experience: recommendations for researchers” in Handbook of emotion elicitation and assessment series in affective science. eds. Coan J. A. Allen J. J. B. (New York, NY, US: Oxford University Press), 361–375.

[ref80] Norris C. J. (2021). The negativity bias, revisited: evidence from neuroscience measures and an individual differences approach. Soc. Neurosci. 16, 68–82. doi: 10.1080/17470919.2019.1696225, PMID: 31750790

[ref81] Ogawa Y. Nittono H. (2019a). Effects of induced moods on semantic memory activation in single-word imagery processing. doi: 10.31234/osf.io/5jerd 31158398

[ref82] Ogawa Y. Nittono H. (2019b). The effect of induced mood on word imagery processing: an ERP study. Int. J. Psychophysiol. 142, 17–24. doi: 10.1016/j.ijpsycho.2019.05.010, PMID: 31158398

[ref83] Out C. Goudbeek M. Krahmer E. (2020). Do Speaker’s emotions influence their language production? Studying the influence of disgust and amusement on alignment in interactive reference. Lang. Sci. 78:101255. doi: 10.1016/j.langsci.2019.101255

[ref84] Pace-Schott E. F. Amole M. C. Aue T. Balconi M. Bylsma L. M. Critchley H.. (2019). Physiological feelings. Neurosci. Biobehav. Rev. 103, 267–304. doi: 10.1016/j.neubiorev.2019.05.002, PMID: 31125635

[ref85] Pinheiro A. P. del Re E. Nestor P. G. McCarley R. W. Gonçalves Ó. F. Niznikiewicz M. (2013). Interactions between mood and the structure of semantic memory: event-related potentials evidence. Soc. Cogn. Affect. Neurosci. 8, 579–594. doi: 10.1093/scan/nss035, PMID: 22434931 PMC3682442

[ref86] Pratt N. L. Kelly S. D. (2008). Emotional states influence the neural processing of affective language. Soc. Neurosci. 3, 434–442. doi: 10.1080/17470910802188339, PMID: 18633830

[ref88] Richards C. Bouman W. P. Seal L. Barker M. J. Nieder T. O. T’Sjoen G. (2016). Non-binary or genderqueer genders. Int. Rev. Psychiatry 28, 95–102. doi: 10.3109/09540261.2015.1106446, PMID: 26753630

[ref89] Rottenberg J. Kovacs M. Yaroslavsky I. (2018). Non-response to sad mood induction: implications for emotion research. Cogn Emot 32, 431–436. doi: 10.1080/02699931.2017.1321527, PMID: 28466682 PMC6174537

[ref90] Rottenberg J. Ray R. D. Gross J. J. (2007). “Emotion elicitation using films” in Handbook of emotion elicitation and assessment series in affective science. eds. Coan J. A. Allen J. J. B. (New York, NY, US: Oxford University Press), 9–28.

[ref91] Rowe G. Hirsh J. B. Anderson A. K. (2006). Positive affect increases the breadth of attentional selection. PNAS 104, 383–388. doi: 10.1073/pnas.0605198104, PMID: 17182749 PMC1765470

[ref92] Russell J. A. (1980). A circumplex model of affect. J. Pers. Soc. Psychol. 39, 1161–1178. doi: 10.1037/h0077714, PMID: 36166650

[ref93] Sakaki M. Gorlick M. A. Mather M. (2011). Differential interference effects of negative emotional states on subsequent semantic and perceptual processing. Emotion 11, 1263–1278. doi: 10.1037/a0026329, PMID: 22142207 PMC3342928

[ref95] Schwarz N. (1990). “Feelings as information: informational and motivational functions of affective states” in Handbook of motivation and cognition: Foundations of social behavior, vol. 2. eds. Higgins E. T. Sorrentino R. M. (New York, NY, US: The Guilford Press), 527–561.

[ref96] Schwarz N. (2002). “Situated cognition and the wisdom in feelings: cognitive tuning” in The wisdom in feeling: Psychological processes in emotional intelligence emotions and social behavior. eds. Barrett L. F. Salovey P. (New York, NY, US: The Guilford Press), 144–166.

[ref97] Schwarz N. Clore G. L. (1983). Mood, misattribution, and judgments of well-being: informative and directive functions of affective states. J. Pers. Soc. Psychol. 45, 513–523. doi: 10.1037/0022-3514.45.3.513, PMID: 34978262

[ref98] Scollon C. N. Diener E. Oishi S. Biswas-Diener R. (2005). An experience sampling and cross-cultural investigation of the relation between pleasant and unpleasant affect. Cognit. Emot. 19, 27–52. doi: 10.1080/02699930441000076

[ref99] Scrimin S. Mason L. (2015). Does mood influence text processing and comprehension? Evidence from an eye-movement study. Br. J. Educ. Psychol. 85, 387–406. doi: 10.1111/bjep.12080, PMID: 26010020

[ref100] Shrout P. E. Rodgers J. L. (2018). Psychology, science, and knowledge construction: broadening perspectives from the replication crisis. Annu. Rev. Psychol. 69, 487–510. doi: 10.1146/annurev-psych-122216-011845, PMID: 29300688

[ref101] Spotorno N. Cheylus A. Henst J.-B. V. D. Noveck I. A. (2013). What’s behind a P600? Integration operations during irony processing. PLoS One 8:e66839. doi: 10.1371/journal.pone.0066839, PMID: 23826155 PMC3691266

[ref102] Sterenberg Mahon A. J. Roth E. A. (2022). What elicits music-evoked nostalgia? An exploratory study among college students. Psychol. Music. 213–222. doi: 10.1177/03057356221087446

[ref103] Stone A. A. Schneider S. Harter J. K. (2012). Day-of-week mood patterns in the United States: on the existence of ‘blue Monday’, ‘thank god it’s Friday’ and weekend effects. J. Posit. Psychol. 7, 306–314. doi: 10.1080/17439760.2012.691980

[ref104] Storbeck J. Clore G. L. (2008). The affective regulation of cognitive priming. Emotion 8, 208–215. doi: 10.1037/1528-3542.8.2.208, PMID: 18410195 PMC2376275

[ref105] Unkelbach C. Forgas J. P. Denson T. F. (2008). The turban effect: the influence of Muslim headgear and induced affect on aggressive responses in the shooter bias paradigm. J. Exp. Soc. Psychol. 44, 1409–1413. doi: 10.1016/j.jesp.2008.04.003

[ref106] Van Berkum J. J. A. (2019). Language comprehension and emotion. The Oxford Handbook of Neurolinguistics. 35, 871–876. doi: 10.1080/23273798.2019.1665191

[ref107] Van Berkum J. J. A. De Goede D. Van Alphen P. M. Mulder E. R. Kerstholt J. H. (2013). How robust is the language architecture? The case of mood. Front. Psychol. 4:505. doi: 10.3389/fpsyg.2013.00505 23986725 PMC3749370

[ref108] Van Berkum J. J. A. Rueschemeyer S. Gaskell G. (2018). “Language comprehension, emotion, and sociality: Aren’t we missing something?” in The Oxford Handbook of Psycholinguistics. eds. Rueschemeyer S. Gareth Gaskell M. 2nd edn. 644–669. doi: 10.1093/oxfordhb/9780198786825.013.28

[ref109] Verhees M. W. F. T. Chwilla D. J. Tromp J. Vissers C. T. W. M. (2015). Contributions of emotional state and attention to the processing of syntactic agreement errors: Evidence from P600. Front. Psychol. 6:388. doi: 10.3389/fpsyg.2015.00388 25914660 PMC4391228

[ref110] Vissers C. T. W. M. Chwilla U. G. Egger J. I. M. Chwilla D. J. (2013). The interplay between mood and language comprehension: evidence from P600 to semantic reversal anomalies. Neuropsychologia 51, 1027–1039. doi: 10.1016/j.neuropsychologia.2013.02.007, PMID: 23454263

[ref111] Vissers C. T. W. M. Virgillito D. Fitzgerald D. A. Speckens A. E. M. Tendolkar I. Van Oostrom I.. (2010). The influence of mood on the processing of syntactic anomalies: evidence from P600. Neuropsychologia 48, 3521–3531. doi: 10.1016/j.neuropsychologia.2010.08.001, PMID: 20696180

[ref112] Wang L. Zhou B. Zhou W. Yang Y. (2016). Odor-induced mood state modulates language comprehension by affecting processing strategies. Sci. Rep. 6:36229. doi: 10.1038/srep36229, PMID: 27796356 PMC5087082

[ref115] Werner-Seidler A. Moulds M. L. (2012). Mood repair and processing mode in depression. Emotion 12, 470–478. doi: 10.1037/a0025984, PMID: 22023367

[ref116] Westermann R. Spies K. Stahl G. Hesse F. W. (1996). Relative effectiveness and validity of mood induction procedures: a meta-analysis. Eur. J. Soc. Psychol. 26, 557–580. doi: 10.1002/(SICI)1099-0992(199607)26:4<557::AID-EJSP769>3.0.CO;2-4

[ref118] Wu Y. J. Thierry G. (2012). How Reading in a second language protects your heart. J. Neurosci. 32, 6485–6489. doi: 10.1523/JNEUROSCI.6119-11.2012, PMID: 22573670 PMC6621108

[ref120] Yano M. Suzuki Y. Koizumi M. (2018). The effect of emotional state on the processing of Morphosyntactic and semantic reversal anomalies in Japanese: evidence from event-related brain potentials. J. Psycholinguist. Res. 47, 261–277. doi: 10.1007/s10936-017-9528-5, PMID: 29119313

[ref121] Zadra J. Clore G. (2011). Emotion and perception: the role of affective information. Wiley interdisciplinary reviews. Cognitive science 2, 676–685. doi: 10.1002/wcs.147, PMID: 22039565 PMC3203022

